# Supplementary material for: Scaling read aligners to hundreds of threads on general-purpose processors
Source: Bioinformatics. 2018 Jul 18;35(3):421–32. doi: 10.1093/bioinformatics/bty648 (PMC6361242; doi:10.1093/bioinformatics/bty648)
Supplement: Supplementary Notes [file bty648_supplementary_notes.pdf]

Supplement for: Scaling read aligners to hundreds of threads on general-purpose processors

Ben Langmead[1,2], Christopher Wilks[1,2], Valentin Antonescu[1], and Rone Charles[1]

[1]Department of Computer Science, Johns Hopkins University

[2]Center for Computational Biology, Johns Hopkins University

June 11, 2018

Supplementary Note 1   Software versions tested

On the Broadwell system, all software was built with GCC version 5.1.0 and the TBB library used was version 4.3. On the KNL system, all software was built with GCC 5.4.0 and the TBB library used was version 2017.0.

Bowtie

- For experiments using original parsing (O) and original `TinyThread++` lock type:
  - Source code: `baseline_paper_v2` tag from Bowtie repo:

    `https://github.com/BenLangmead/bowtie`
  - Compile-time preprocessor macros:

    `WITH_THREAD_PROFILING=1`

    `EXTRA_FLAGS="-DUSE_FINE_TIMER"`
  - Alignment-time command-line parameters:

    `-I 250 -X 800`
- For experiments using original parsing (O) and *TBB standard* lock type:
  - Source code: same as for, `TinyThread++` lock type above.
  - Compile-time preprocessor macros:

    `WITH_THREAD_PROFILING=1`

    `EXTRA_FLAGS="-DUSE_FINE_TIMER"`

    `WITH_TBB=1`

    `NO_SPINLOCK=1`
  - Alignment-time command-line parameters: same as for `TinyThread++` lock type above.
- For experiments using original parsing (O) and TBB *spin* lock type:
  - Source code: same as for, `TinyThread++` lock type above.

- Compile-time preprocessor macros:

  `WITH_THREAD_PROFILING=1`

  `EXTRA_FLAGS="-DUSE_FINE_TIMER"`

  `WITH_TBB=1`

- Alignment-time command-line parameters: same as for `TinyThread++` lock type above.

- For experiments using original parsing (O) and TBB *queueing* lock type:
  - Source code: same as for `TinyThread++` lock type above.
  - Compile-time preprocessor macros:

    `WITH_THREAD_PROFILING=1`

    `EXTRA_FLAGS="-DUSE_FINE_TIMER"`

    `WITH_TBB=1`

    `NO_SPINLOCK=1`

    `WITH_QUEUELOCK=1`

  - Alignment-time command-line parameters: same as for `TinyThread++` lock type above.

- For deferred parsing (D) experiments:
  - Source code: `parsing_paper_v2` tag from Bowtie repo:

    `https://github.com/BenLangmead/bowtie`

  - Compile-time preprocessor macros:

    `WITH_THREAD_PROFILING=1`

    `EXTRA_FLAGS="-DUSE_FINE_TIMER"`

    `WITH_TBB=1`

    `NO_SPINLOCK=1`

    `WITH_QUEUELOCK=1`

  - Alignment-time command-line parameters:

`-I 250 -X 800`

`--reads-per-batch 1`

- For deferred batch parsing (B) experiments:
  - Source code: same as for deferred parsing (D) above.
  - Compile-time preprocessor macros: same as for deferred parsing (D) above.
  - Alignment-time command-line parameters:

    `-I 250 -X 800`

    `--reads-per-batch 32`

- For deferred batch parsing with separate input/output thread (B*) experiments:
  - Source code: `queue` tag from Bowtie repo:
  - Compile-time preprocessor macros: same as for batch parsing (B) above.
  - Alignment-time command-line parameters:

    `-I 250 -X 800`

    `--reads-per-batch 32`

- For MP baseline experiments:
  - Source code: same as for deferred batch parsing (B) above.
  - Compile-time preprocessor macros: same as for deferred batch parsing (B) above.
  - Alignment-time command-line parameters:

    `-I 250 -X 800`

    `--reads-per-batch 32`

    `--mm`

- For blocked-FASTQ experiments:
  - Source code: `blocked_paper_v2` tag from Bowtie repo:

    `https://github.com/BenLangmead/bowtie`

  - Compile-time preprocessor macros: same as for deferred parsing (D) above.
  - Alignment-time command-line parameters:

    `-I 250 -X 800`

    `--block-bytes 12288`

    `--reads-per-block 70` for unpaired, or:

    `--reads-per-block 44` for paired-end

- For blocked-FASTQ experiments with striped output:
  - Source code: `blocked_striped_paper_v2` tag from Bowtie repo:

    `https://github.com/BenLangmead/bowtie`

  - Compile-time preprocessor macros: same as for deferred parsing (D) above.
  - Alignment-time command-line parameters:

    `-I 250 -X 800`

    `--block-bytes 12288`

    `--reads-per-block 70` for unpaired, or:

    `--reads-per-block 44` for paired-end

Bowtie 2

- For experiments using original parsing (O) and original `TinyThread++` lock type:
  - Source code: `baseline_paper_v1` tag from Bowtie 2 repo:

    `https://github.com/BenLangmead/bowtie2`

  - Compile-time preprocessor macros:

    `WITH_THREAD_PROFILING=1`

    `EXTRA_FLAGS="-DUSE_FINE_TIMER"`

  - Alignment-time command-line parameters:

    `-I 250 -X 800`

- For experiments using original parsing (O) and *TBB standard* lock type:

  - Source code: same as for `TinyThread++` lock type above.
  - Compile-time preprocessor macros:

    ```
    WITH_THREAD_PROFILING=1
    EXTRA_FLAGS="-DUSE_FINE_TIMER"
    WITH_TBB=1
    NO_SPINLOCK=1
    ```

  - Alignment-time command-line parameters: same as for `TinyThread++` lock type above.

- For experiments using original parsing (O) and TBB *spin* lock type:

  - Source code: same as for `TinyThread++` lock type above.
  - Compile-time preprocessor macros:

    ```
    WITH_THREAD_PROFILING=1
    EXTRA_FLAGS="-DUSE_FINE_TIMER"
    WITH_TBB=1
    ```

  - Alignment-time command-line parameters: same as for `TinyThread++` lock type above.

- For experiments using original parsing (O) and TBB *queueing* lock type:

  - Source code: same as for `TinyThread++` lock type above.
  - Compile-time preprocessor macros:

    ```
    WITH_THREAD_PROFILING=1
    EXTRA_FLAGS="-DUSE_FINE_TIMER"
    WITH_TBB=1
    NO_SPINLOCK=1
    WITH_QUEUELOCK=1
    ```

  - Alignment-time command-line parameters: same as for `TinyThread++` lock type above.

- For deferred parsing (D) experiments:
  - Source code: `parsing_paper_v2` tag from Bowtie 2 repo:

    `https://github.com/BenLangmead/bowtie2`
  - Compile-time preprocessor macros:

    ```
    WITH_THREAD_PROFILING=1
    EXTRA_FLAGS="-DUSE_FINE_TIMER"
    WITH_TBB=1
    NO_SPINLOCK=1
    WITH_QUEUELOCK=1
    ```
  - Alignment-time command-line parameters:

    ```
    -I 250 -X 800
    --reads-per-batch 1
    ```
- For deferred batch parsing (B) experiments:
  - Source code: same as for deferred parsing (D) above.
  - Compile-time preprocessor macros: same as for deferred parsing (D) above.
  - Alignment-time command-line parameters:

    ```
    -I 250 -X 800
    --reads-per-batch 32
    ```
- For MP baseline experiments:
  - Source code: same as for deferred batch parsing (B) above.
  - Compile-time preprocessor macros: same as for deferred batch parsing (B) above.
  - Alignment-time command-line parameters:

    ```
    -I 250 -X 800
    --reads-per-batch 32
    --mm
    ```

- For blocked-FASTQ experiments:
  - Source code: `blocked_paper_v2` tag from Bowtie 2 repo:

    `https://github.com/BenLangmead/bowtie2`
  - Compile-time preprocessor macros: same as for deferred parsing (D) above.
  - Alignment-time command-line parameters:

    `-I 250 -X 800`

    `--block-bytes 12288`

    `--reads-per-block 70` for unpaired, or:

    `--reads-per-block 44` for paired-end
- For blocked-FASTQ experiments with striped output:
  - Source code: `blocked_striped_paper_v2` tag from Bowtie 2 repo:

    `https://github.com/BenLangmead/bowtie2`
  - Compile-time preprocessor macros: same as for deferred parsing (D) above.
  - Alignment-time command-line parameters:

    `-I 250 -X 800`

    `--block-bytes 12288`

    `--reads-per-block 70` for unpaired, or:

    `--reads-per-block 44` for paired-end

HISAT

- For experiments using original parsing (O) and original `TinyThread++` lock type:
  - Source code: `baseline_paper_v1` tag from the following fork of the HISAT repo:

    `https://github.com/BenLangmead/hisat`
  - Compile-time preprocessor macros:

    `WITH_THREAD_PROFILING=1`

`EXTRA_FLAGS="-DUSE_FINE_TIMER"`

  - Alignment-time command-line parameters:

    `-I 250 -X 800`

    `--no-spliced-alignment`

    `--no-temp-splicesite`

- For experiments using original parsing (O) and *TBB standard* lock type:
  - Source code: same as for `TinyThread++` lock type above.
  - Compile-time preprocessor macros:

    `WITH_THREAD_PROFILING=1`

    `EXTRA_FLAGS="-DUSE_FINE_TIMER"`

    `WITH_TBB=1`

    `NO_SPINLOCK=1`

  - Alignment-time command-line parameters: same as for `TinyThread++` lock type above.
- For experiments using original parsing (O) and TBB *spin* lock type:
  - Source code: same as for `TinyThread++` lock type above.
  - Compile-time preprocessor macros:

    `WITH_THREAD_PROFILING=1`

    `EXTRA_FLAGS="-DUSE_FINE_TIMER"`

    `WITH_TBB=1`

  - Alignment-time command-line parameters: same as for `TinyThread++` lock type above.
- For experiments using original parsing (O) and TBB *queueing* lock type:
  - Source code: same as for `TinyThread++` lock type above.
  - Compile-time preprocessor macros:

    `WITH_THREAD_PROFILING=1`

```
EXTRA_FLAGS="-DUSE_FINE_TIMER"
WITH_TBB=1
NO_SPINLOCK=1
WITH_QUEUELOCK=1
```

- Alignment-time command-line parameters: same as for `TinyThread++` lock type above.

- For deferred parsing (D) experiments:
  - Source code: `parsing_paper_v2` tag from the following fork of the HISAT repo:

    `https://github.com/BenLangmead/hisat`
  - Compile-time preprocessor macros:

```
WITH_THREAD_PROFILING=1
EXTRA_FLAGS="-DUSE_FINE_TIMER"
WITH_TBB=1
NO_SPINLOCK=1
WITH_QUEUELOCK=1
```

  - Alignment-time command-line parameters:

```
-I 250 -X 800
--reads-per-batch 1
--no-spliced-alignment
--no-temp-splicesite
```

- For deferred batch parsing (B) experiments:
  - Source code: same as for deferred parsing (D) above.
  - Compile-time preprocessor macros: same as for deferred parsing (D) above.
  - Alignment-time command-line parameters:

```
-I 250 -X 800
```

`--reads-per-batch 32`

`--no-spliced-alignment`

`--no-temp-splicesite`

- For MP baseline experiments:
  - Source code: same as for deferred batch parsing (B) above.
  - Compile-time preprocessor macros: same as for deferred batch parsing (B) above.
  - Alignment-time command-line parameters:

    `-I 250 -X 800`

    `--reads-per-batch 32`

    `--no-spliced-alignment`

    `--no-temp-splicesite`

    `--mm`

- For blocked-FASTQ experiments:
  - Source code: `blocked_paper_v2` tag from the following fork of the HISAT repo:

    `https://github.com/BenLangmead/hisat`
  - Compile-time preprocessor macros: same as for deferred parsing (D) above.
  - Alignment-time command-line parameters:

    `-I 250 -X 800`

    `--no-spliced-alignment`

    `--no-temp-splicesite`

    `--block-bytes 12288`

    `--reads-per-block 70` for unpaired, or:

    `--reads-per-block 44` for paired-end

- For blocked-FASTQ experiments with striped output:

- Source code: `blocked_striped_paper_v2` tag from the following fork of the HISAT repo:

  `https://github.com/BenLangmead/hisat`

- Compile-time preprocessor macros: same as for deferred parsing (D) above.
- Alignment-time command-line parameters:

  `-I 250 -X 800`

  `--no-spliced-alignment`

  `--no-temp-splicesite`

  `--block-bytes 12288`

  `--reads-per-block 70` for unpaired, or:

  `--reads-per-block 44` for paired-end

BWA-MEM

- For all experiments:
  - Source code: `chunksz_oflow` branch of the following fork of the `bwa` repo:

    `https://github.com/BenLangmead/bwa`

  - Compile-time preprocessor macros: none
  - Alignment-time command-line parameters. Note that BWA-MEM estimates the fragment length distribution from a sample of the data.

Supplementary Note 2 Read set download links

- human_100_300M
  - http://www.cs.jhu.edu/~langmea/resources/mix100_1.fq.gz
  - http://www.cs.jhu.edu/~langmea/resources/mix100_2.fq.gz
- human_50_300M
  - http://www.cs.jhu.edu/~langmea/resources/mix50_1.fq.gz
  - http://www.cs.jhu.edu/~langmea/resources/mix50_2.fq.gz
- human_100_block_300M
  - http://www.cs.jhu.edu/~langmea/resources/mix100_block_1.fq.gz
  - http://www.cs.jhu.edu/~langmea/resources/mix100_block_2.fq.gz
- human_50_block_300M
  - http://www.cs.jhu.edu/~langmea/resources/mix50_block_1.fq.gz
  - http://www.cs.jhu.edu/~langmea/resources/mix50_block_2.fq.gz

Supplementary Note 3 BWA-MEM chunk size fix

We noticed an issue in the BWA-MEM source code that would create issues for experiments using more than 214 threads (`-t 215` or greater). Specifically, a signed 32-bit integer is used to represent the "chunk size," i.e. the number of input bases to include in a single chunk. That number is 10 million by default, but it is multiplied by the thread count to calculate the final chunk size. This multiplication results in integer overflow for thread counts greater than 214, leading to behavior that dramatically slowed down read alignment. We fixed this issue and submitted a pull request to the BWA-MEM author. The pull request is located at https://github.com/lh3/bwa/pull/155. The work was originally done in the `chunksz_oflow` branch of our BWA fork: https://github.com/BenLangmead/bwa/tree/chunksz_oflow.

	Broadwell		KNL	
	Unpaired	Paired	Unpaired	Paired
Bowtie	1,000,000	110,000	450,000	37,500
Bowtie 2	200,000	85,000	65,000	16,000
BWA-MEM	200,000	85,000	65,000	16,000
HISAT	1,200,000	550,000	400,000	250,000

Supplementary Table 1: Number of reads per thread for all the experiments. These were chosen to ensure that all runs take about a minute or longer.

		Skylake (96 threads)						Broadwell (112 threads)						KNL (272 threads)					
		Paired			Unpaired			Paired			Unpaired			Paired			Unpaired		
		Th	Krd/s	Whole human (mins)	Th	Krd/s	Whole human (mins)	Th	Krd/s	Whole human (mins)	Th	Krd/s	Whole human (mins)	Th	Krd/s	Whole human (mins)	Th	Krd/s	Whole human (mins)
Bowtie	O-parsing, TinyThread++ spin	96	129.83	154.05	24	465.01	43.01	88	98.69	202.64	24	282.93	70.69	72	41.96	476.67	32	127.48	156.89
	O-parsing, TBB spin	96	132.85	150.54	16	385.84	51.84	88	97.01	206.17	16	229.62	87.10	72	42.92	465.98	24	118.74	168.44
	O-parsing, TBB standard	80	122.40	163.40	32	427.26	46.81	72	86.08	232.35	24	239.80	83.40	48	26.54	753.61	16	81.67	244.90
	O-parsing, TBB queueing	92	132.74	150.67	40	584.61	34.21	96	103.61	193.03	48	415.78	48.10	72	39.22	509.99	48	123.33	162.17
	D-parsing	96	143.62	139.26	40	697.17	28.69	104	102.51	195.11	32	264.94	75.49	136	63.55	314.71	56	173.14	115.52
	B-parsing	96	148.28	134.88	48	1,080.64	18.51	112	115.41	173.29	40	556.38	35.95	192	67.14	297.88	104	269.28	74.27
	L-parsing	92	145.17	137.77	96	1,634.77	12.23	112	114.69	174.39	104	1,216.74	16.44	144	65.60	304.88	232	702.22	28.48
	L-parsing, 2 outputs	96	141.63	141.21	96	1,705.03	11.73	108	115.01	173.89	104	1,325.77	15.09	192	65.78	304.05	268	852.33	23.47
	L-parsing, 4 outputs	96	141.46	141.38	96	1,765.78	11.33	112	116.45	171.75	104	1,270.46	15.74	136	65.87	303.61	264	875.69	22.84
	L-parsing, 8 outputs	96	141.86	140.98	96	1,790.44	11.17	112	116.13	172.21	104	1,201.42	16.65	136	65.43	305.69	264	1,060.14	18.87
	L-parsing, 16 outputs	96	141.95	140.89	96	1,794.86	11.14	112	115.64	172.95	100	1,317.21	15.18	184	65.13	307.07	268	1,104.62	18.11
	MP baseline	96	130.01	153.84	96	1,433.97	13.95	112	108.89	183.68	112	1,215.75	16.45	272	70.46	283.83	272	884.69	22.61
Bowtie 2	O-parsing, TinyThread++ spin	88	65.88	303.57	64	168.18	118.92	80	51.55	387.99	80	125.02	159.97	96	16.49	1,212.94	96	43.71	457.54
	O-parsing, TBB spin	88	67.82	294.90	80	175.63	113.88	64	55.45	360.69	72	137.07	145.91	96	18.17	1,100.87	88	47.09	424.73
	O-parsing, TBB standard	56	62.44	320.30	72	161.26	124.03	56	49.05	407.76	64	124.19	161.04	64	12.73	1,570.74	64	36.36	550.09
	O-parsing, TBB queueing	80	66.72	299.76	88	175.56	113.92	64	54.48	367.07	88	144.14	138.75	96	17.72	1,128.42	88	46.64	428.77
	D-parsing	92	73.21	273.20	92	189.25	105.68	104	58.93	339.38	100	159.18	125.64	268	27.21	735.12	216	65.10	307.22
	B-parsing	96	73.25	273.05	96	193.83	103.18	104	58.41	342.42	104	161.77	123.64	264	26.53	753.97	268	72.20	277.02
	L-parsing	96	73.22	273.16	96	193.96	103.12	96	56.74	352.48	104	156.48	127.81	272	26.27	761.43	272	72.12	277.30
	L-parsing, 2 outputs	96	71.94	278.02	96	198.99	100.51	100	58.51	341.83	100	160.73	124.43	268	26.32	759.80	268	71.92	278.10
	L-parsing, 4 outputs	96	71.98	277.84	96	198.29	100.86	108	58.87	339.73	104	164.57	121.53	272	26.40	757.67	272	72.03	277.67
	L-parsing, 8 outputs	96	71.82	278.48	96	199.17	100.42	112	59.06	338.62	104	162.82	122.83	272	26.41	757.17	272	72.10	277.40
	L-parsing, 16 outputs	96	71.70	278.94	96	198.50	100.76	108	58.84	339.88	104	160.21	124.84	272	26.25	761.82	272	72.01	277.73
	MP baseline	96	67.85	294.79	96	179.77	111.26	112	57.11	350.21	112	159.47	125.42	272	27.94	715.86	272	70.76	282.66
	BWA-MEM	92	60.98	327.99	88	163.77	122.13	104	49.92	400.65	104	134.07	149.18	232	19.93	1,003.52	232	50.01	399.93
HISAT	O-parsing, TinyThread++ spin	16	137.72	145.22	16	297.79	67.16	16	89.80	222.71	16	210.14	95.17	16	29.11	686.94	24	71.72	278.87
	O-parsing, TBB spin	16	138.82	144.07	16	308.40	64.85	16	94.10	212.54	16	209.14	95.63	16	29.55	676.89	16	72.18	277.09
	O-parsing, TBB standard	16	95.87	208.61	24	267.85	74.67	16	61.89	323.14	16	162.16	123.33	8	19.96	1,002.19	12	43.60	458.74
	O-parsing, TBB queueing	16	135.42	147.68	24	336.07	59.51	16	98.45	203.15	32	238.34	83.91	32	27.65	723.38	24	72.56	275.64
	D-parsing	48	344.21	58.10	48	648.73	30.83	108	144.26	138.64	108	230.70	86.69	64	95.46	209.52	64	179.68	111.31
	B-parsing	56	543.45	36.80	56	1,089.28	18.36	56	315.24	63.44	40	540.92	36.97	64	148.32	134.84	64	288.79	69.25
	L-parsing	92	737.63	27.11	96	1,449.77	13.80	96	571.25	35.01	108	1,033.25	19.36	136	255.61	78.24	128	468.96	42.65
	L-parsing, 2 outputs	96	786.31	25.44	92	1,579.81	12.66	104	563.34	35.50	104	1,180.38	16.94	240	318.91	62.71	224	606.59	32.97
	L-parsing, 4 outputs	96	796.54	25.11	92	1,625.66	12.30	104	569.43	35.12	100	1,169.80	17.10	268	375.94	53.20	268	703.83	28.42
	L-parsing, 8 outputs	96	800.10	25.00	96	1,670.97	11.97	104	580.66	34.44	104	1,214.55	16.47	268	390.61	51.20	268	791.19	25.28
	L-parsing, 16 outputs	96	802.14	24.93	96	1,677.32	11.92	104	566.02	35.33	100	1,212.91	16.49	268	398.04	50.25	268	828.71	24.13
	MP baseline	96	706.14	28.32	96	1,484.40	13.47	112	568.00	35.21	112	1,154.60	17.32	192	298.34	67.04	240	675.67	29.60

Supplementary Table 2: Peak throughputs for all experimental series, including multiprocessing baselines and BWA-MEM. Each row reports: maximal peak throughput in thousands of reads per second ("Krd/s"), number of threads that achieved the peak ("Th"), and the number of wall-clock minutes required to align a 40-fold coverage dataset assuming 100 nt reads (or 100 x 100 pairs for paired-end scenarios) and a 3-billion bp genome ("Whole human"). For each combination of aligner, paired-end status and test system, the best and second-best throughputs are highlighted red and orange respectively. B-parsing and BWA-MEM rows are highlighted as those represent the default modes for those tools.

Input set	Uncompressed			Gzipped		
	FQ (GB)	Blocked FQ (GB)	Relative change	FQ (GB)	Blocked FQ (GB)	Relative change
`human_100_300M` end 1	76.86	83.78	+9.01%	28.47	28.77	+1.02%
`human_100_300M` both ends	153.72	167.56	+9.01%	57.00	57.57	+1.00%
`human_50_300M` end 1	46.26	52.66	+13.84%	16.01	16.25	+1.47%
`human_50_300M` both ends	92.52	105.32	+13.84%	32.39	32.87	+1.45%

Supplementary Table 3: Impact of block padding on FASTQ file size. File sizes are in gigabytes.

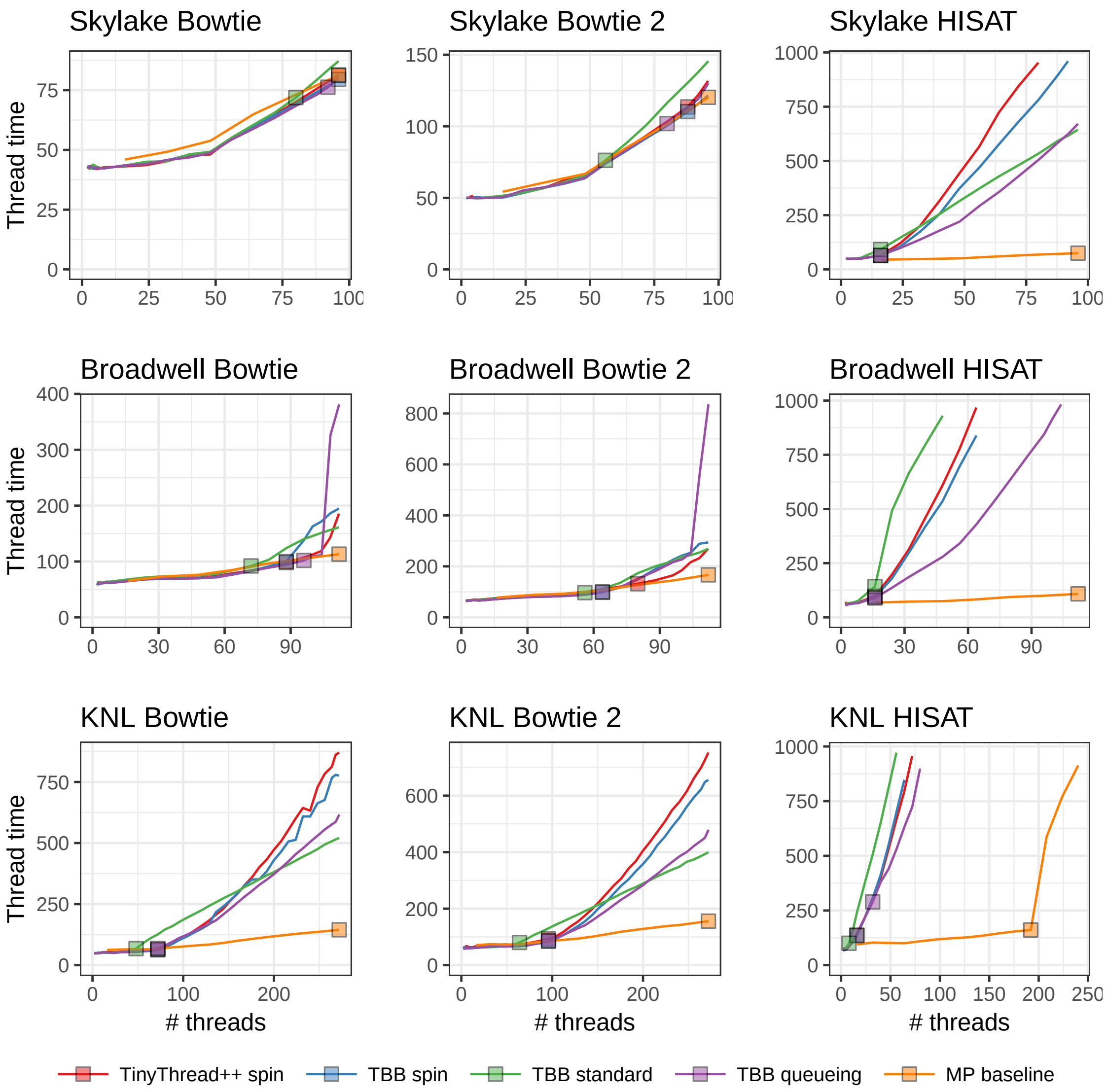

Supplementary Figure 1: Comparison of 4 lock types and multiprocessing baseline. Reads are paired-end. Results are shown for three aligners (rows) and two systems (columns). Jobs that ran for over 20 minutes are omitted. Squares indicate the point on each line yielding maximal total alignment throughput. These are summarized in Table 2 in the main text.

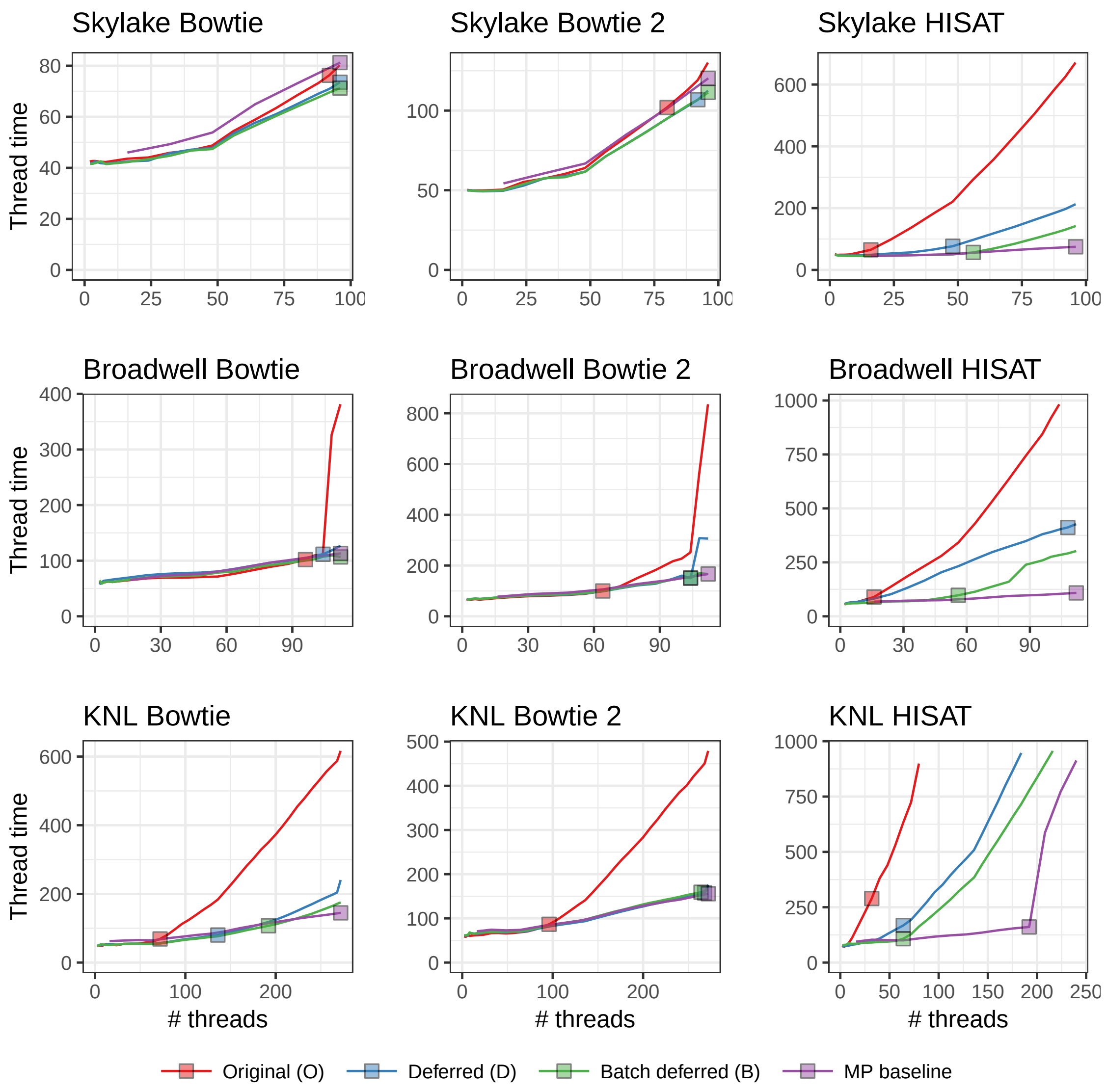

Supplementary Figure 2: Comparison of 3 parsing strategies and multiprocessing baseline. Reads are paired-end. Jobs that ran for over 20 minutes are omitted. Squares indicate the point on each line yielding maximal total alignment throughput and these points are summarized in Table 3 in the main text.

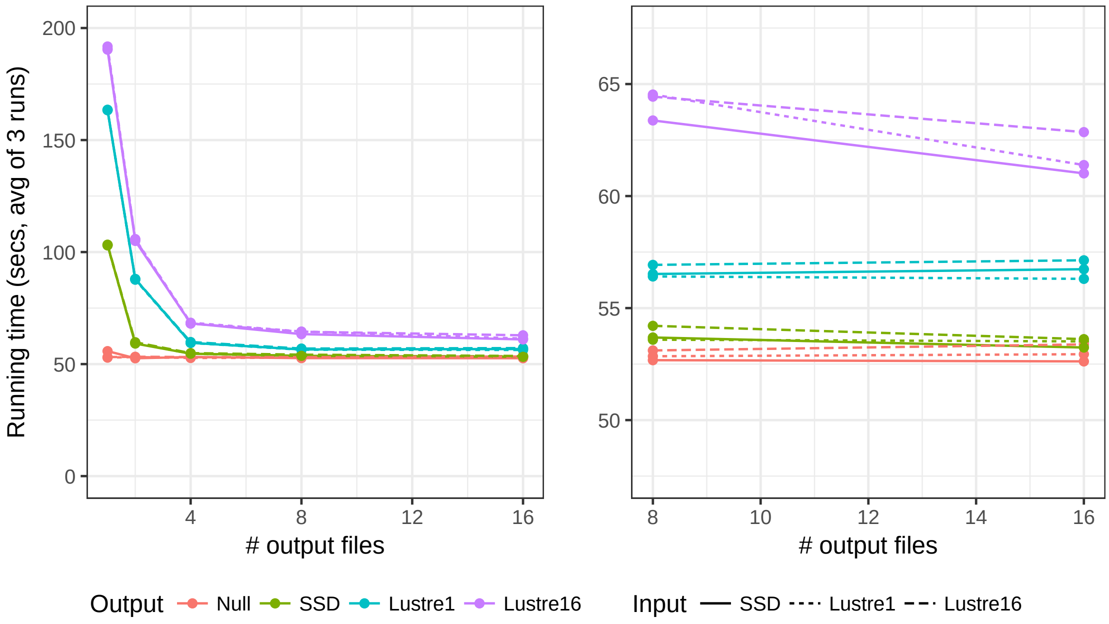

Supplementary Figure 3: HISAT running time versus number of striped output files on KNL. Input and output filesystems were varied; "SSD" is a local solid-state drive, "Lustre1" is a directory on a Lustre system set to use 1 stripe, "Lustre16" is a directory on a Lustre system set to use 16 stripes. "Null" means output is directed to `/dev/null`. Left plot shows full results, while right plot zooms in on the 8- and 16-file results. The first 16M pairs from the `human_100_block_300M` input set were aligned. The results indicate that running time depends much more on output filesystem than on input filesystem. Increasing the number of output files reduces running time up to 16 files. This is likely because striping reduces contention for the output lock(s). It is the output striping that achieves the gain; increasing the number of stripes using Lustre's own striping mechanism seems only to reduce throughput. Even at 16 output-file stripes, there is a notable difference in running time depending on output file system, indicating that output can be a bottleneck for fast and scalable aligners

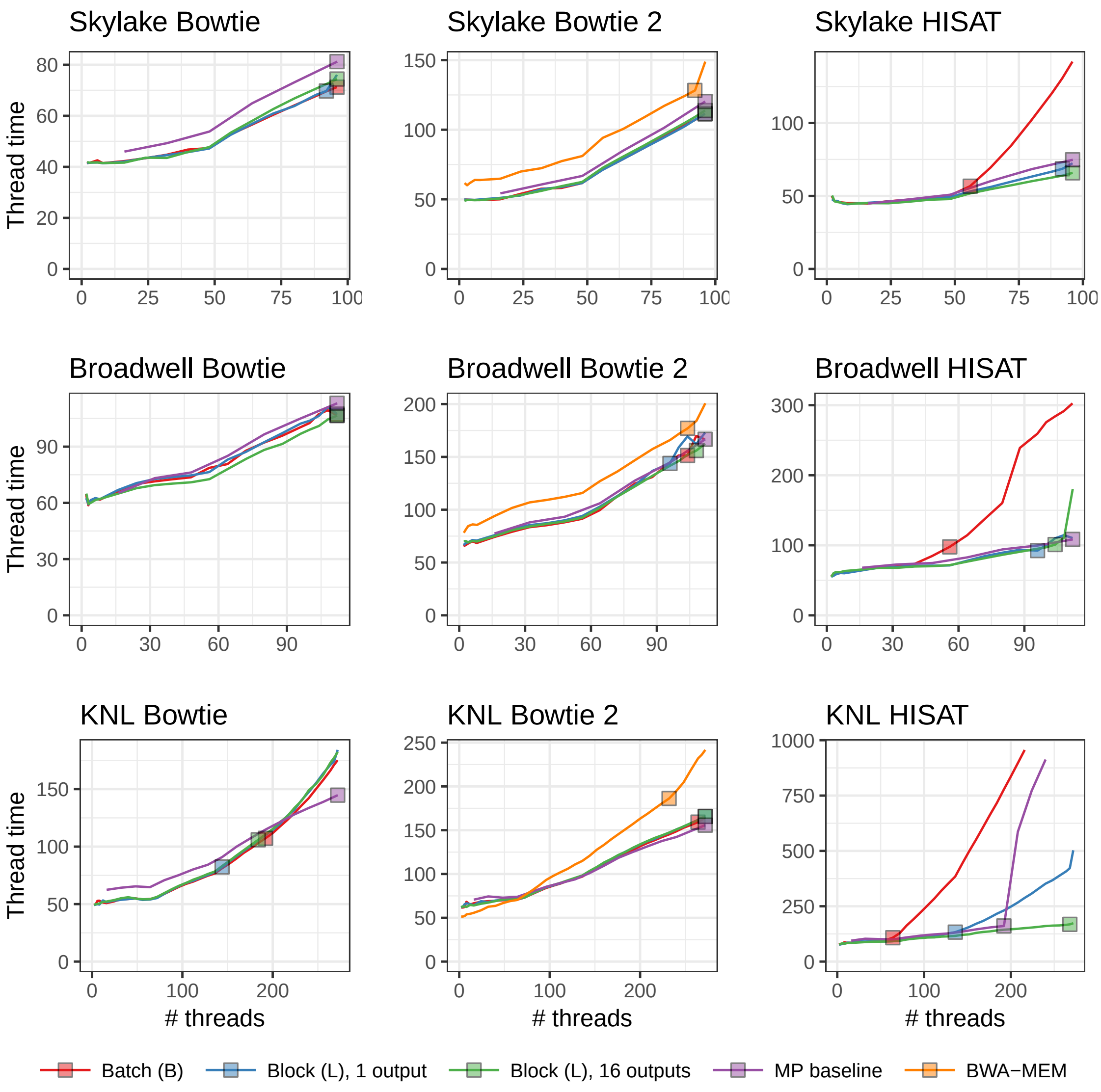

Supplementary Figure 4: Paired-end-alignment comparison of B-parsing, L-parsing, L-parsing with output striped across 16 files and the MP baseline. BWA-MEM is also evaluated and compared to the Bowtie 2 configurations. Jobs that ran for over 20 minutes are omitted. Squares indicate the run for each configuration yielding greatest overall alignment throughput, also summarized in Table 4 in the main text.

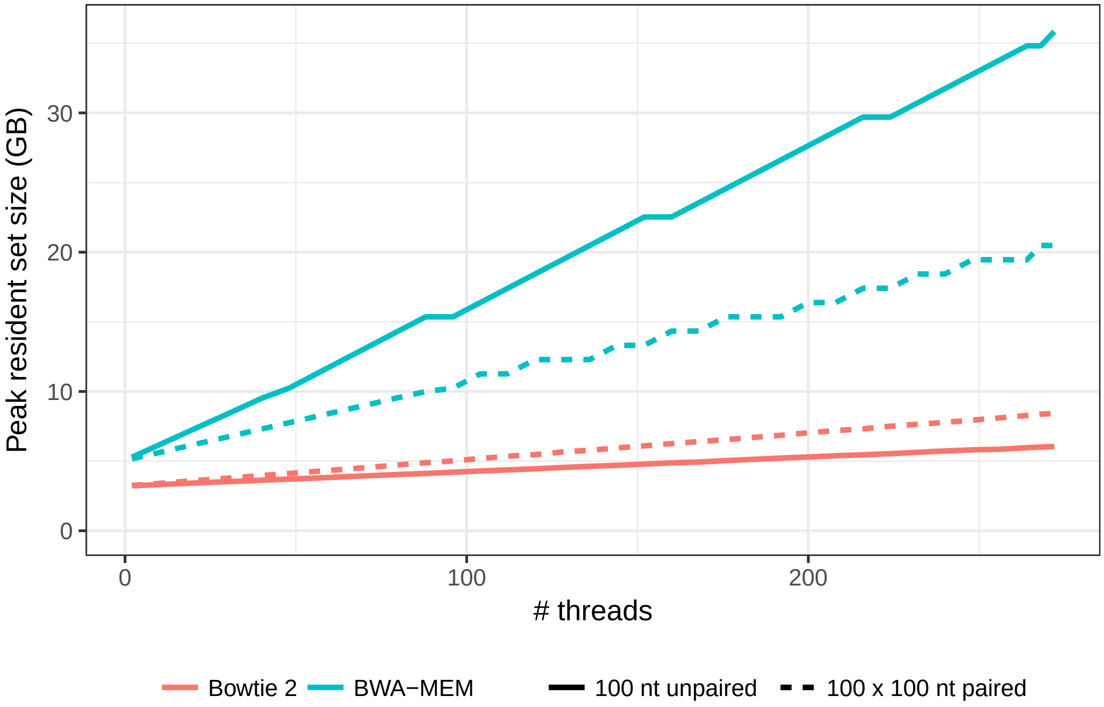

Supplementary Figure 5: BWA-MEM's peak resident set size (memory footprint) grows more quickly than Bowtie 2's on KNL. This is likely due to BWA-MEM's large batch size and the fact that the batch size scales linearly with the number of threads.

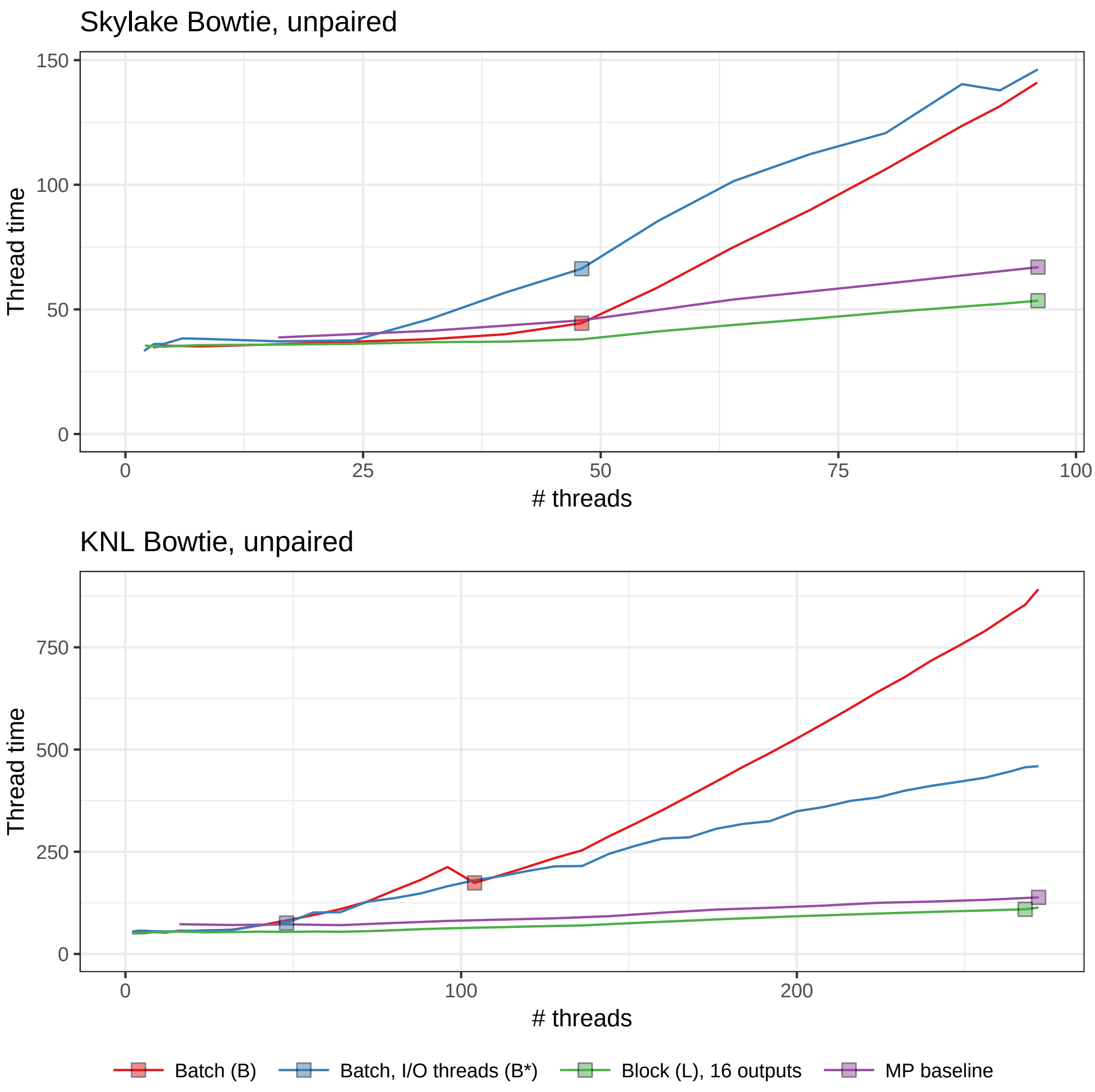

Supplementary Figure 6: Comparison of B-parsing, output-striped L-parsing, the MP baseline, and a version of B-parsing that uses separate threads for input and output parsing, called B*-parsing here. Input and output threads exchange records with the worker threads via queues. The "Moody Camel" multi-producer multi-consumer queue is used. Only unpaired alignment using Bowtie is assessed.
